# Systematic review of digital interventions to support refusal self-efficacy in child and adolescent health promotion

**DOI:** 10.1093/heapro/daac085

**Published:** 2022-09-27

**Authors:** Johanna Nyman, Anna Tornivuori, Sanna Salanterä, Teresa Barroso, Heidi Parisod

**Affiliations:** Department of Nursing Science, FI-20014, University of Turku; Department of Nursing Science, FI-20014, University of Turku; Turku University Hospital, Health Village, Turku, Finland; Department of Nursing Science, FI-20014, University of Turku; Turku University Hospital, Turku, Finland; Nursing School of Coimbra, Rua 5 de Outubro, Apartado 7001, 3046-851 Coimbra, Portugal; Department of Nursing Science, FI-20014, University of Turku; Nursing Research Foundation sr (NRF), Asemamiehenkatu 2, 00520 Helsinki, Finland

**Keywords:** child, adolescent, health behavior, health promotion, digital health intervention, self-efficacy, systematic review

## Abstract

Refusal self-efficacy protects against risky health behavior. Digital interventions have the potential to support self-efficacy due to the enactive experience provided by digital technologies. The aim of this systematic literature review was to evaluate the evidence of digital interventions to support refusal self-efficacy in child and adolescent health promotion. Following the Cochrane Collaboration guidelines, five electronic databases were searched from 2009 to 2020. The studies were assessed by two independent reviewers according to the eligibility criteria. Eligible studies were included in the review, assessed for risk of bias, synthesized narratively and assessed for evidence quality with the GRADE approach. Twenty-three studies, that examined 18 different interventions, were included in the review. The interventions included various digital elements as means to support the child and adolescent refusal self-efficacy (e.g. games, videos, feedback and activities for regulating feelings). The interventions improving refusal self-efficacy were more often used at home setting and addressed the four sources of self-efficacy with different digital elements regardless of intervention duration and intensity. Although the results on intervention effects varied and the evidence quality remained low, the overall evidence concerning these interventions was encouraging. Based on the subgroup analysis, the results were mainly encouraging among girls. When these interventions are implemented in health promotion, their benefits and weaknesses need to be considered comprehensively. The results provide information for designing and developing digital interventions to support child and adolescent refusal self-efficacy. Further research with larger sample sizes and more rigorous study designs is needed to strengthen the evidence of these interventions.

## INTRODUCTION

When approaching adolescence, children begin to explore and adopt new health behaviors (Viner *et al.*, 2012). Supporting self-efficacy promotes health-related behavior and intentions (Sheeran *et al.*, 2016). Self-efficacy deals with judgements of own capacities (Bandura, 1997), and is defined as a belief in own ability to perform necessary behavior or activities to achieve a desired goal, thus playing a central role in personal agency and decision-making (Bandura, 1977). Self-efficacy beliefs are based on four sources of information—mastery experiences (experiences of success or failure), vicarious experiences (modeling and social comparison), social persuasion (feedback and support) and physiological and emotional states—which are cognitively processed and reflected before being integrated into self-efficacy judgements (Bandura, 1997). Refusal self-efficacy has been derived from the concept of self-efficacy, and it is defined as a belief in own ability to refuse or resist for example substance use (Oei *et al.*, 1998; Oei and Morawska, 2004) or unwanted sex (Zimmermann *et al.*, 1995; Vanable *et al.*, 2009).

Refusal self-efficacy, among some other factors, determines child and adolescent health behavior in different domains of health: substance abuse, smoking, healthy nutrition and sexual behavior (Peters *et al.*, 2009). Research indicates that refusal self-efficacy influences both positive and negative health behaviors. It serves directly as a protective factor against adolescent risky health behavior (for example, substance use, smoking and sexual experience) (Jang *et al.* 2013; Wang *et al.*, 2019) and also moderates the negative influence of descriptive social norms related to peers’ risky health behavior (Jang *et al.* 2013). It has been suggested that a decrease in adolescent refusal self-efficacy is related significantly to adopting negative health behavior (Hiemstra *et al.*, 2011).

In child and adolescent (later child) health promotion, there is a need for innovative and effective interventions that develop refusal self-efficacy (Jang *et al.*, 2013; McKelvey *et al.*, 2014; Wang *et al.*, 2019) to help them overcome social and emotional pressures to adopt risky health behaviors (Bandura, 2004). Digital technologies and environments offer new possibilities for child health promotion since their use of digital technologies is prevalent (Anderson and Jiang, 2018). In the field of health, the use of digital technologies as well as information and communication technologies to improve health is referred to as digital health (World Health Organization [WHO], 2016; European Commission, 2022). Digital interventions can be used in different domains of health and healthcare. They have great potential for improving health by providing effective, safe and cost-effective tools (Murray *et al.*, 2016.) For example, the enactive experience and observational learning elements afforded by digital games provide a good means to support children’s health-related self-efficacy by enabling mastery experiences, vicarious experiences (Lee, 2015) as well as social persuasion (Pakarinen *et al.*, 2017).

Synthesized information from different studies concerning the evidence and elements of digital interventions is needed for evaluating and further developing the interventions (Murray *et al.*, 2016) and for informing clinical practice. An increasing number of digital interventions to promote children’s health have been developed and evaluated for effectiveness. However, to our knowledge, reviews concerning digital interventions that support refusal self-efficacy in child health promotion are lacking. According to a literature review, refusal self-efficacy (but not, for example, general self-efficacy) is one of the determinants that is relevant for at least four health behaviors in adolescence, and thus, needs to be considered in health promotion interventions (Peters *et al.*, 2009). To understand the phenomenon of refusal self-efficacy more broadly and to be able to further develop digital interventions to support it, the aim of this systematic review was to evaluate the evidence of digital interventions to support refusal self-efficacy in child health promotion. This review also aims at advancing the use of effective interventions in child health promotion.

## METHODS

This systematic review was conducted following the Cochrane Collaboration guidelines for conducting (Higgins and Green, 2011) as well as the Preferred Reporting Items for Systematic Reviews and Meta-Analyses (PRISMA) standards for reporting systematic reviews (Moher *et al.*, 2009). The protocol was registered with the PROSPERO database (registration number: CRD42019127775).

### Search strategy

A systematic search of published literature was performed in March 2019 and updated in April 2020 to reach the most recent studies. PubMed/Medline, CINAHL, Cochrane Library, PsycINFO and Embase databases were searched from 2009 to April 2020. This time frame was chosen since technology evolves rapidly requiring digital interventions to be repeatedly developed (Michie *et al.*, 2017) and updated (Murray *et al.*, 2016).

An information specialist was consulted in forming the search strings. Search terms including subject terms and word truncation were used. The search string comprised of relevant terms describing digital interventions, children, refusal self-efficacy as well as experimental or quasi-experimental study design. Different types of health behavior interventions were accepted for our study since the main focus was on child refusal self-efficacy and not on the different health behaviors. For example, the search string for PubMed/Medline database was as follows: (digital* OR video* OR electronic* OR interactive* OR online* OR computer* OR internet* OR social media* OR smartphone* OR game* OR gamif* OR virtual reality* OR console* OR mobile* OR tablet* OR multimedia* OR application* OR app OR apps OR software*) AND (intervention OR program* OR “Health Promotion”[Mesh] OR “Program Evaluation”[Mesh]) AND (adolescen* OR teen* OR preadolescen* OR preteen* OR mid-adolescen* OR mid-teen* OR child* OR youth* OR youngster* OR young people* OR young person* OR “Adolescent”[Mesh] OR “Child”[Mesh] OR “Minors”[Mesh]) AND (anti OR refus* OR non OR prevent* OR avoid* OR declin*) AND (self-efficacy [tiab] OR efficacy expectation* [tiab] OR “Self Efficacy”[Mesh]) AND (RCT* OR experiment* OR quasi-experiment* OR clinical trial* OR trial* OR randomi* OR randomly OR pre-post OR pretest-posttest OR pre/post). No other filters were used than those concerning publication date. In addition to the electronic database search, the reference lists of relevant publications were searched manually.

### Eligibility criteria

The studies that met the following eligibility criteria were included in the review:

### Participants

The majority (≥ 50 %) of participants are children or adolescents up to the age of 18.

### Intervention

Digital health interventions that are in a virtual form and aim to support refusal self-efficacy and promote health.

### Comparator

Both intervention and control conditions enable evaluating the effectiveness.

### Outcome

Refusal self-efficacy was measured with instruments before and after the intervention.

### Study design

Experimental or quasi-experimental study design.

### Study selection

After performing the searches, all the duplicates were removed. Titles of all the remaining records were screened by one reviewer based on the inclusion criteria, and all potentially relevant publications were included for abstract screening. All publications that were potentially relevant were finally assessed based on the full-text. After full-text examination, all publications that met the eligibility criteria were included in the review and all included publications were reviewed by two independent reviewers. The full-text assessments were mainly consistent but there were a few discrepancies related to the definitions of digital intervention and refusal self-efficacy which were solved to resolved.

### Assessment of risk of bias

The risk of bias was assessed by two independent reviewers using the Cochrane Collaboration’s tool for assessing the risk of bias based on the following criteria: random sequence generation (selection bias), allocation concealment (selection bias), blinding of participants and personnel (performance bias), blinding of outcome assessment (detection bias), blinding of outcome assessment (detection bias), incomplete outcome data addressed (attrition bias), and selective reporting (reporting bias) (Higgins *et al.*, 2011).

### Data extraction and synthesis

All relevant data were extracted based on a predetermined data extraction plan. The data extraction plan was formulated based on the review questions. The Cochrane Collaboration’s checklist of items to consider in data collection or data extraction (Higgins and Deeks, 2011) and the template for intervention description and replication (TIDieR) checklist (Hoffman *et al.*, 2014) were adapted for the data extraction plan. Data concerning the study (including authors, publication details, design and aim of the study, methods, measured outcomes and results), the intervention (including intervention name, materials, provider, modes of delivery, setting and duration and intensity), as well as the manifestation of refusal self-efficacy in the intervention (including the theoretical background and assumptions, perspective, measures and results) were extracted from each study included in the review. When necessary, the descriptions of the interventions were expanded on by examining publications (Romer *et al.*, 2009; Tortolero *et al.*, 2010; Cremers *et al.*, 2012; Fang and Schinke, 2013; Rundle-Thiele *et al.*, 2013; Parisod *et al.*, 2017; Winskell *et al.*, 2019) where these interventions have been described in more detail. Data concerning the characteristics of the studies and the interventions, the manifestation of refusal self-efficacy, and the intervention effects were synthesized narratively. Deductive content analysis was applied when analyzing the manifestation of refusal self-efficacy in the interventions. The categorization matrix was developed from the sources of self-efficacy in Bandura’s (1997) theory, and the digital elements of the interventions were identified in the studies and coded according to the categories (Elo and Kyngäs, 2008). In addition, the intervention effects could be summarized in a table (see Table 1). In this table, we compare the *p*-values of each study on refusal self-efficacy with the digital elements used to support refusal self-efficacy.

**Table 1.** Effects of the digital health interventions on child and adolescent refusal self-efficacy based on the included digital elements and the addressed sources of self-efficacy.

	Mastery experiences					Vicarious experiences				Social persuasion				Emotions and physiological states				
	Games with skill-based activities	Exercises to practice and try out skills	Role-play activities	Goal and limit setting exercises	Quizzes to reinforce knowledge	Peer modeling videos, stories, and voice messages	Animated scenes demonstrating consequences	Role-models introducing activities	Chat forums with peers	Feedback on tasks	Incentives, scores, points, and prizes	Advancing in intervention based on choices	Online counselling and support	Supporting positive feelings and communication	Platform for reflection and personalization	Feel-good material	Activities for regulating and coping with feelings	Intervention effects (+/ 0)
1. Schinke *et al.* (Schinke *et al.*, 2009)			X		X			X			X	X		X				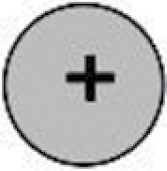
2. Fang and Schinke (Fang and Schinke, 2013, 2014)	X	X	X	X	X		X					X					X	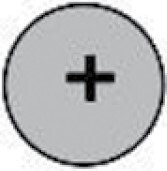
3. Fang *et al.* (Fang *et al.*, 2010)	X	X	X	X			X					X						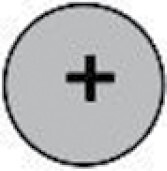
4. Norris *et al.* (Norris *et al.*, 2013)	X										X							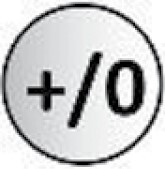
5. Chang *et al*. (Chang et al., 2018, 2019)						X					X		X	X				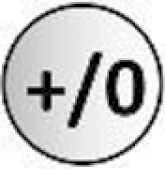
6. Parisod *et al.* (Parisod *et al.*, 2018)	X				X		X				X			X				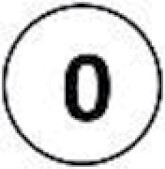
7. Cremers *et al.* (Cremers *et al.*, 2015)	X					X				X			X					NA
8. Dietrich *et al.* (Dietrich *et al.*, 2015)	X					X												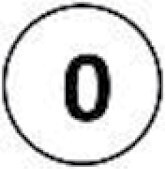
9. Peskin *et al.* (Peskin *et al.*, 2019)					X	X				X					X			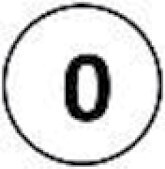
10. Potter *et al.* (Potter *et al.*, 2016)					X	X				X					X			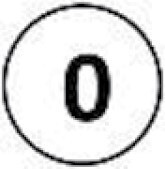
11. Tortolero *et al*. (Tortolero *et al.*, 2010)					X	X				X					X			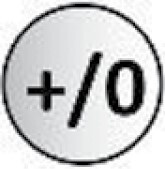
12. Peskin *et al.* (Peskin *et al.*, 2015)		X	X			X									X			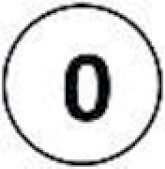
13. Dcruz (Dcruz, 2014)		X		X											X		X	NA
14. Ismayilova and Terlikbayeva (Ismayilova and Terlikbayeva, 2018)	X	X			X	X		X					X					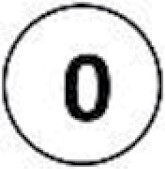
15. Sznitman *et al*. (Sznitman *et al.*, 2011)						X								X		X		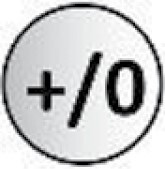
16. Kaufman *et al.* (Kaufman *et al.*, 2018)	X					X												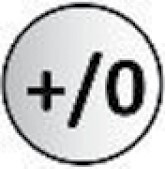
17. Lotrean *et al.* (Lotrean *et al.*, 2010)						X		X		X								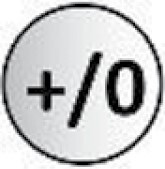
18. Schwinn *et al*. (Schwinn *et al.*, 2010)				X				X	X		X				X	X		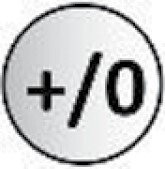
19. Cunningham *et al*. (Cunningham *et al.*, 2009)			X				X	X		X								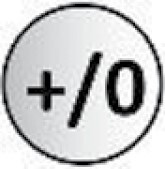
20. Markham *et al.* (Markham *et al.*, 2012)		X				X				X					X			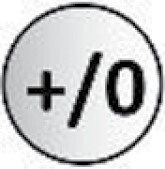
21. Winskell *et al.* (Winskell *et al.*, 2018)	X	X	X	X	X		X			X	X		X				X	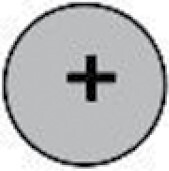
22. Musiimenta (Musiimenta, 2012)			X		X	X		X					X					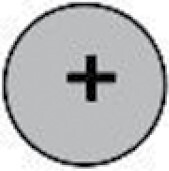

+, statistically significant favorable results on refusal self-efficacy; 0, statistically nonsignificant results on refusal self-efficacy; +/0, both statistically significant favorable results and statistically nonsignificant results on refusal self-efficacy at different time points, with different subgroups, or domains of refusal self-efficacy. NA, not available.

The Grading of Recommendations Assessment, Development, and Evaluation (GRADE) approach was applied to evaluate the quality of the evidence of the refusal self-efficacy outcomes based on five criteria: study limitations (risk of bias related to the study design and execution), inconsistency of results (unexplained heterogeneity), indirectness of evidence (indirect comparisons or differences in population, interventions or outcome measures), imprecision (in the estimates of effect), and publication bias (selective study publication). Although the GRADE approach is most applicable to meta-analyses, it has been used to evaluate the evidence quality of systematic reviews. (Schünemann *et al.*, 2013.)

## RESULTS

**Fig. 1:**
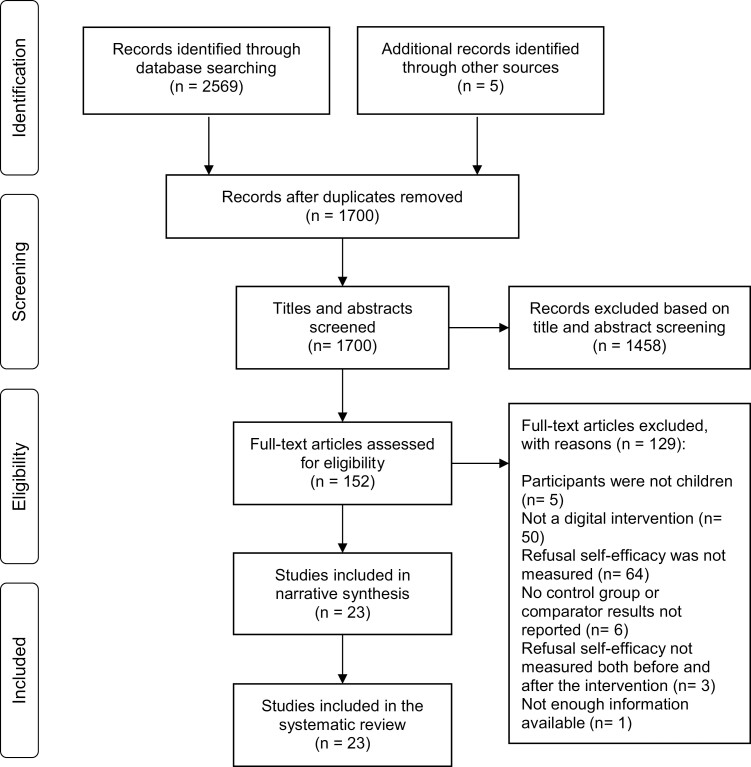
Study selection presented with PRISMA flow diagram (Moher *et al.*, 2009).

Altogether 23 studies were included in this review, see in detail in Figure 1. A meta-analysis could not be performed due to the heterogeneity of the instruments measuring refusal self-efficacy in the reviewed studies.

### Risk of bias of the included studies

The risk of bias assessment is presented in a supplementary file for each domain of risk of bias and for each individual study (see Supplementary Table S1). The risk of bias in most of the studies remained unclear due to the lack of detailed reporting. For example, although almost all studies (*n* = 22) reported having used randomization, only eight studies reported how randomization was done (Lotrean *et al.*, 2010; Fang and Schinke, 2013; Cremers *et al.*, 2015; Potter *et al.*, 2016; Chang *et al.*, 2018; Kaufman *et al.*, 2018; Parisod *et al.*, 2018; Winskell *et al.*, 2018). Only two studies reported allocation concealment (Fang and Schinke, 2013; Winskell *et al.*, 2018) and one blinding outcome assessment (Fang and Schinke, 2013). However, with a small research group, blinding of outcome assessment might be challenging to arrange. Most of the studies reported no blinding of participants and personnel, and those which did, reported only blinding of participants or personnel instead of reporting blinding of both. Outcome data (both short- and longer-term) were reported rather well. There were only four studies in which this attrition bias was assessed as high risk mainly due to uneven attrition (Sznitman *et al.*, 2011; Peskin *et al.*, 2015; Potter *et al.*, 2016). Reporting bias was assessed as having the overall lowest risk of bias since all but two studies reported their pre-specified outcomes (Dcruz, 2014; Cremers *et al.*, 2015). The last criterion, other bias, was assessed rather often as having high or unclear risk mostly due to differences between the study groups. Only four studies were assessed as having a low risk of other bias (Lotrean *et al.*, 2010; Fang and Schinke, 2013; Chang *et al.*, 2018; Ismayilova and Terlikbayeva, 2018).

Due to the predominantly unclear risk of bias in the selected studies, the synthesis of the effects of the digital interventions needs to be treated with caution. However, it does not influence the general description of the digital interventions or how refusal self-efficacy was manifested in these interventions.

### Characteristics of the included studies

The characteristics of the included studies are summarized in a supplementary file (Supplementary Table S2). The studies were published during 2009–2019 with 10 studies published during 2015 or after and six studies during 2018 or after. Three studies examined the same intervention (Tortolero *et al.*, 2010; Potter *et al.*, 2016; Peskin *et al.*, 2019). One study examined two different versions of this intervention (Markham *et al.*, 2012), and two studies examined a full computer-based version of it (Dcruz, 2014; Peskin *et al.*, 2015). In addition, another three studies examined the one intervention with two of them studying the same data (Fang *et al.*, 2010; Fang and Schinke, 2014, 2013).

### Characteristics of the digital interventions

There were 18 interventions examined in the 23 studies, and over half of the interventions had a specific name (see Supplementary Table S2). The health topics covered in these interventions were mainly sexual health (*n* = 10) and substance use (*n* = 6), alcohol (*n* = 3) and smoking (*n* = 2) prevention.

Most of the interventions (*n* = 16) were delivered by computers or online. Two interventions were delivered by smartphones or tablets (Parisod *et al.*, 2018; Winskell *et al.*, 2018) and one by television or radio (Sznitman *et al.*, 2011). Although all the interventions were assessed as mainly digital, the interventions also included some non-digital elements, such as group activities in the school environment (Lotrean *et al.*, 2010; Tortolero *et al.*, 2010; Markham *et al.*, 2012; Norris *et al.*, 2013; Potter *et al.*, 2016; Peskin *et al.*, 2019), skills practice activities (Tortolero *et al.*, 2010; Dietrich *et al.*, 2015), instructions or demonstrations (Kaufman *et al.*, 2018), and worksheets or homework (Tortolero *et al.*, 2010; Markham *et al.*, 2012; Chang *et al.*, 2018; Peskin *et al.*, 2019).

The duration and intensity of the interventions varied. For example, one completely digital intervention was a 35-min brief intervention (Cunningham *et al.*, 2009) whereas one partly digital intervention was held over two years consisting of 24 lessons of 45–50 min each (Tortolero *et al.*, 2010; Potter *et al.*, 2016). In general, the duration and intensity of the intervention were more stable with interventions that were only partially digital and thus usually guided by trained teachers, assistants or research staff members. In some completely digital interventions, the duration and intensity depended partly on how much the children or adolescents used or were exposed to the intervention, for example, digital games (Parisod *et al.*, 2018; Winskell *et al.*, 2018), a website (Cremers *et al.*, 2015) or media messages (Sznitman *et al.*, 2011).

### Manifestation of refusal self-efficacy in the digital interventions

The interventions were mostly based on the theoretical frameworks of social cognitive theories (*n* = 10), such as Bandura’s social cognitive theory, the theory of planned behavior, the social learning theory, and the theory of reasoned action. Also, other theoretical frameworks that acknowledge the role of self-efficacy in influencing human behavior were used, for example, the family interaction theory or social influence models (see Supplementary Table S2).

**Fig. 2:**
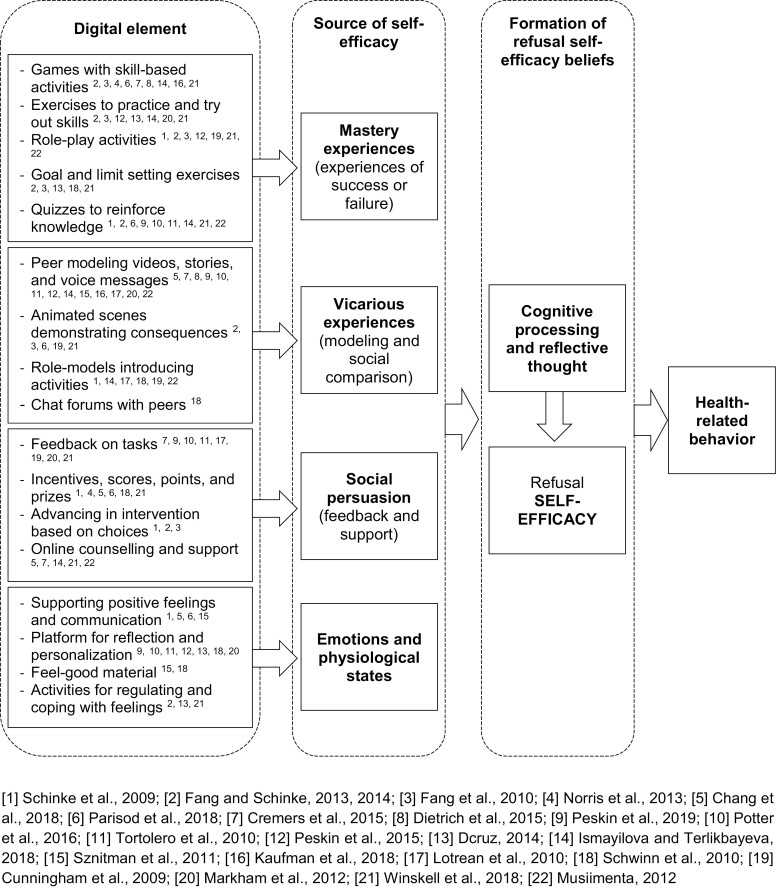
Manifestation of refusal self-efficacy in the digital health interventions according to Bandura’s self-efficacy theory (Bandura, 1997).

The interventions included various different digital elements as means to support the refusal self-efficacy of children and adolescents. These means can be classified into the four sources of self-efficacy, that is, mastery experiences, vicarious experiences, social persuasion as well as physiological and emotional arousal (see Figure 2).

Mastery experiences were provided in these interventions by offering skill-based activities or activities in which the children faced different, often challenging, situations similar to real-world situations. These activities were offered to the children mainly in the form of digital games, role-plays, or quizzes. The interventions addressed vicarious experiences by providing scenes (via videos, stories, voice messages or animated scenes) in which other children or avatars representing them faced challenging situations and demonstrated their skills, and faced different consequences based on their behavior. Social persuasion was addressed in the interventions by providing encouraging feedback on tasks and exercises. The children could also earn points, scores, prices or incentives based on the decision they make in a game or other activity. Elements that acknowledged the regulation of emotions and physiological states emphasized the positive sides of healthy behaviors, provided activities for regulating or coping with feelings, or provided a platform for reflection and personalization.

### Effects of the digital interventions on refusal self-efficacy

The effects of the digital interventions on child refusal self-efficacy varied with six studies showing only statistically significantly favorable results and six studies showing statistically nonsignificant results and others showing both statistically significant and nonsignificant results at different time points (see Table 1). Out of the six studies showing only statistically significant results, two (Fang and Schinke, 2013, 2014) were based on the same data and thus considered as one in the analysis of the intervention effects. In two studies (Dcruz, 2014; Cremers *et al.*, 2015) the effects of the intervention on refusal self-efficacy could not be summarized since the intervention effects on refusal self-efficacy were not reported. In most of the studies, health behavior or behavioral intention was considered as one of the primary outcomes, and in half of the studies self-efficacy was one of the primary outcomes. Also, other outcomes were measured, for example, social norms, attitudes, and knowledge. Due to the different instruments to measure refusal self-efficacy, only a summary description of the intervention effects could be done.

Based on our analysis of the intervention effects, the digital interventions that improved child refusal self-efficacy were mostly used in home settings (*n*/*N* = 4/5), and either addressed to support only girl’s refusal self-efficacy or refusal self-efficacy outcomes were measured only from girls (*n*/*N* = 4/5) (see Supplementary Table S2). These interventions addressed almost all four sources of self-efficacy, especially mastery experiences, with various different digital elements (see Table 1).

In our analysis with the GRADE approach, the main outcome refusal self-efficacy was divided into three subtypes: substance use refusal self-efficacy, sex refusal self-efficacy and peer resistance self-efficacy (see Table 2). More topic-related subtype analysis (for example, based on the substance) could not be done because there were too few studies. The GRADE assessment was made separately for each subtype. The evidence quality was assessed as low for substance use refusal self-efficacy and sex refusal self-efficacy, and very low for peer resistance self-efficacy. Since there was heterogeneity in the results, a subgroup analysis based on gender was made (Schünemann *et al.*, 2013). In this analysis, the evidence quality of digital interventions to support girls’ substance use refusal self-efficacy was assessed as moderate. A similar analysis could not be done for boys, because there were no studies examining only boys’ refusal self-efficacy. There was heterogeneity in the age of the participants, but a subgroup analysis could not be done because age-based results on refusal self-efficacy were not reported in most of the studies and the age range of the participants varied across the studies. Also, the duration of follow-up varied across the studies, but the results showed no clear direction based on it. Since the duration of follow-up and the interventions varied greatly, a subgroup analysis for different measurement points could not be done.

**Table 2:** GRADE evidence profile: digital interventions to support child and adolescent refusal self-efficacy.

Quality assessment							Summary of findings^a^				Quality	Importance
No. of studies	Study design	Study limitations	Inconsistency	Indirectness	Imprecision	Publication bias	No. of participants		Effect			
							Intervention group	Control group	Relative (95 % CI)	Absolute (95 % CI)		
Substance use refusal self-efficacy (measured with different instruments, follow-up range from 0–2 weeks to 25 months)												
10^b^	RCT and cluster RCT	No serious limitations	Serious inconsistency ^c^	No serious indirectness	Serious imprecision ^d^	No serious publication bias	1815	1886	–	Not estimable	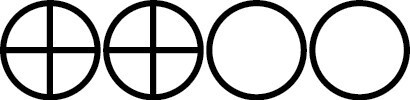 Low	Critical
Sex refusal self-efficacy (measured with different instruments, follow-up range from 0–1 week to 26 months)												
8^e^	RCT, cluster RCT and non-randomized experimental trial	Serious limitations ^f^	Serious inconsistency ^c^	No serious indirectness	No serious imprecision	No serious publication bias	4465	4320	–	Not estimable	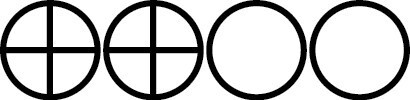 Low	Critical
Peer resistance self-efficacy (measured with different instruments, follow-up ranged from 0–2 weeks to 9 months)												
2^g^	RCT and cluster RCT	Serious limitations ^f^	No serious inconsistency	No serious indirectness	Very serious imprecision^h^	No serious publication bias	62	62	–	Not estimable	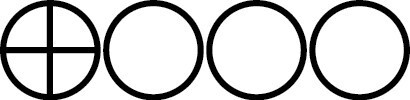 Very low	Critical

Study-specific findings are presented in a supplementary file (Supplementary Table S3).

Schinke *et al.* (Schinke *et al.*, 2009), Fang and Schinke (Fang and Schinke, 2013, 2014), Fang *et al*. (Fang *et al.*, 2010), Chang *et al*. (Chang *et al.*, 2018), Parisod *et al.* (Parisod *et al.*, 2018), Dietrich *et al*. (Dietrich *et al.*,2015), Ismayilova and Terlikbayeva (Ismayilova and Terlikbayeva, 2018), Lotrean *et al.* (Lotrean *et al.*, 2010), Schwinn *et al.* (Schwinn *et al.*,2010), Cunningham *et al.* (Cunningham *et al.*,2009).

There was a serious inconsistency in the results (−1). The inconsistency can be explained by differences in populations (gender, risk factors, age), interventions (characteristics, dose and setting) or outcomes (duration of follow-up).

There was a serious imprecision in the results (−1). The imprecision can be explained by small sample sizes.

Peskin *et al.* (Peskin *et al.*,2019), Potter *et al.* (Potter *et al.*, 2016), Tortolero *et al*. (Tortolero *et al.*, 2010), Peskin *et al.* (Peskin *et al.*, 2015), Sznitman *et al.* (Sznitman *et al.*, 2011), Kaufman *et al.* (Kaufman *et al.*, 2018), Markham *et al.* (Markham *et al.*, 2012), Winskell *et al*. (Winskell *et al.*, 2018), Musiimenta (Musiimenta, 2012).

There were serious limitations in the studies (−1). The limitations can be explained by lack of blinding, differential or major loss to follow-up, and imbalance or possible contamination between study conditions.

Norris *et al.* (Norris *et al.*, 2013) and Kaufman *et al*. (Kaufman *et al.*, 2018).

There was very serious imprecision in the results (-2). The imprecision can be explained by very small sample sizes.

## DISCUSSION

This systematic review describes digital interventions to support refusal self-efficacy in child health promotion as well as the evidence of these interventions. The results suggest that different interventions with several digital elements have been developed and used.

Based on our review, digital elements supporting personal *mastery experiences* and *vicarious experiences* were most commonly used to support child refusal self-efficacy (as in Figure 2 and Table 1). The interventions supporting mastery experiences with different digital elements also reported more often improvements in refusal self-efficacy. This is consistent with Bandura’s self-efficacy theory according to which mastery experiences and vicarious experiences are most effective in developing self-efficacy beliefs since they provide such evidence of one’s capabilities that is the most authentic and easier to judge (Bandura, 1997).

Also, digital elements supporting *social persuasion* as well as *physiological and emotional arousal* were used. This differs from the systematic review of Pakarinen *et al.* (2017) in which emotional and physiological states were not addressed in health game interventions supporting child self-efficacy related to physical activity. According to Bandura (1997), physiological and emotional states are especially relevant in health functioning. Morrison (2015) suggests that digital interventions in the domain of health need to provide positively framed feedback that supports self-efficacy by acquainting with challenges to it. This supports our analysis of intervention effects since it was found that the interventions showing statistically significant favorable results on refusal self-efficacy addressed more often the majority of the four sources of self-efficacy.

According to our review, evaluating the effects of individual digital elements supporting child refusal self-efficacy is challenging. This is because all interventions comprised of more than one digital element or method that addressed the sources of self-efficacy, and the studies examined the effects of whole interventions and not only some parts of them. It was also challenging to evaluate and synthesize the effects of the interventions on refusal self-efficacy since several different measurement tools were used, and all tools were domain-specific, some also study-specific. Because of this, the evidence quality was evaluated based on three subtypes (substance use refusal, sex refusal and peer resistance) of refusal self-efficacy. Although there are common aspects of refusal self-efficacy across different health domains (that is, the confidence in one’s ability to refuse risky or harmful behavior), there is evidence that refusal self-efficacy beliefs differ across domains (Choi *et al.*, 2013). For example, Bandura (2006) has argued that the measurement of self-efficacy needs to be tailored to the functioning and behavior in the specific domain.

The duration and intensity of the interventions examined in this review varied. Based on the review it remained unclear whether it is better to guide children to use the digital interventions (for example, how much or often they should use it) or let them use the interventions freely. According to Michie *et al.* (2017) effective engagement can differ widely depending on the intervention type and context, and cannot be evaluated simply by measuring usage rates but needs a specified description regarding each intervention indicating sufficient engagement to attain desired outcomes. Our review supports this view. For example, both a 35-min brief intervention (Cunningham *et al.* 2009) and a longer nine-module intervention (Fang *et al.*, 2010; Fang and Schinke, 2013, 2014) had positive effects whereas both a two-year school-based intervention (Potter *et al.*, 2016; Peskin *et al.*, 2019) and an intervention consisting of two weeks of free usage (Parisod *et al.*, 2018) had no statistically significant positive effects on refusal self-efficacy.

According to our results, the digital interventions that improved child refusal self-efficacy were mostly used in home settings and used various digital elements to support refusal self-efficacy. Although school-based interventions targeting other health areas have had positive results on self-efficacy (for example, Coffman *et al.*, 2009), the results of this review suggest that there might be a need to extend the interventions also to home settings. This might be more relevant with interventions targeting child refusal self-efficacy, like the interventions in our review, since it enables practicing refusal self-efficacy skills also in environments where peer pressure is less present. For example, Hiemstra et al. (2009, 2011) suggest home-based interventions for pre-adolescents and early adolescents when most of them have not yet experienced risky health behavior. However, it needs to be considered, that some confounding factors we could not identify in our analysis, may have influenced the results in the reviewed interventions.

When interpreting the results of our review, the possible subgroup differences need to be considered. For example, the reviewed interventions were targeted at or studied with children of ages 10 to 18 and from different cultural or ethnic backgrounds. There were also gender differences since most of the reviewed studies that improved refusal self-efficacy were targeted only at girls. Research indicates that there are gender differences in attitudes towards health promotion interventions. For example, Lund *et al.* (2020) discovered that girls had more positive attitudes towards smoking-related rules and restrictions than boys. It is not certain that the reviewed interventions are effective in supporting child refusal self-efficacy despite gender, but the certainty of evidence seems to be stronger for interventions aimed only at girls compared to both genders. Differences in age or cultural background were not detected to explain the differences in study results. The effectiveness of these digital interventions among boys could be improved by integrating gender-specific tailored content into them (Lund *et al.*, 2020).

According to our assessment with the GRADE criteria, the evidence quality of the refusal self-efficacy outcomes was low or very low due to inconsistencies, small sample sizes and study limitations (as presented in Table 2). Due to the differences between the reviewed studies, a meta-analysis could not be done which weakens the evidence quality. Although the quality of evidence remained low, it does not indicate that digital interventions are poor methods to support child refusal self-efficacy. Instead, it indicates that the research evidence is insufficient to make stronger estimates of their effectiveness (Schünemann *et al.*, 2013). In relation to child refusal self-efficacy, the evidence supports their use. A more comprehensive evaluation of the benefits and weaknesses of the digital interventions to support child refusal self-efficacy is needed.

### Limitations

This review has some limitations. Since the included studies, especially the digital interventions and the instruments measuring refusal self-efficacy were heterogeneous, it was not possible to perform a meta-analysis. There were also two studies that met the eligibility criteria but did not report the results concerning refusal self-efficacy making it impossible to include them in the evaluation of intervention effects. One study was included in the review in which two of our authors (HP, SS) had participated (the Fume game is owned by the University of Turku and available to use free of charge). To provide an objective assessment, it was ensured that these two did not participate in the eligibility assessment as well as the evaluation of the quality of their own study. To balance the limitations, the Cochrane Collaboration guidelines for conducting systematic reviews were followed including assessing the risk of bias in the selected studies. In our assessment, the risk of bias remained predominantly unclear. Because of this, the high methodological quality of the studies cannot be ensured which undermines the reliability of our review results. Nevertheless, an adequate amount of studies was included in our review which bolsters the generalizability of the results.

## CONCLUSION

The digital interventions improving refusal self-efficacy were more often used in home setting and addressed the four sources of self-efficacy with different digital elements regardless of intervention duration and intensity. The overall evidence concerning the digital interventions was encouraging, but the results on intervention effects varied and the evidence quality was low. Based on the subgroup analysis the results were mainly encouraging among girls. Although the evidence quality remained low, it does not indicate that digital interventions are poor methods to support child refusal self-efficacy. When these interventions are implemented in health promotion, their benefits and weaknesses need to be considered comprehensively. This review provides information for designing and developing digital health interventions to support child refusal self-efficacy by summarizing how refusal self-efficacy can be addressed with various digital elements. To strengthen the evidence, further research on the effectiveness of these interventions could use larger sample sizes and more rigorous study designs and make more elaborated subgroup analysis, for example, based on the age of the participants. This could improve the quality of evidence of these interventions and their applicability in child health promotion.

## Supplementary Material

daac085_suppl_Supplementary_Table_S1

daac085_suppl_Supplementary_Table_S2

daac085_suppl_Supplementary_Table_S3
