# Supplementary material for: Systematic review of digital interventions to support refusal self-efficacy in child and adolescent health promotion
Source: Health Promot Int. 2022 Sep 27;37(5):daac085. doi: 10.1093/heapro/daac085 (PMC10243864; doi:10.1093/heapro/daac085)
Supplement: daac085_suppl_Supplementary_Table_S1 [file daac085_suppl_supplementary_table_s1.docx]

Table S1. Assessment of risk of bias summary of the included studies.

|  | Random sequence generation (selection bias) | Allocation concealment (selection bias) | Blinding of participants and personnel (performance bias) | Blinding of outcome assessment (detection bias) (patient-reported outcomes) | Incomplete outcome data addressed (attrition bias) (Short-term outcomes (<6 weeks)) | Incomplete outcome data addressed (attrition bias) (Longer-term outcomes (>6 weeks)) | Selective reporting (reporting bias) | Other bias |
| --- | --- | --- | --- | --- | --- | --- | --- | --- |
| Schinke et al., 2009 | 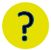 | 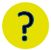 | 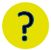 | 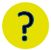 | 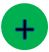 | 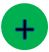 | 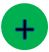 | 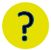 |
| Fang and Schinke, 2014 | 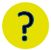 | 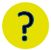 | 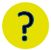 | 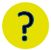 | NA | 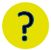 | 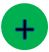 | 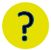 |
| Fang and Schinke, 2013 | 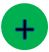 | 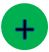 | 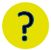 | 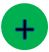 | NA | 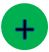 | 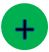 | 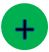 |
| Fang et al., 2010 | 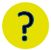 | 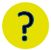 | 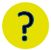 | 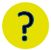 | NA | 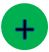 | 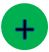 | 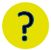 |
| Norris et al., 2013 | 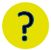 | 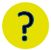 | 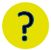 | 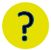 | 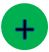 | 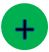 | 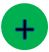 | 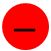 |
| Chang et al., 2018 | 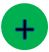 | 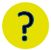 | 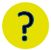 | 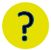 | NA | 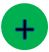 | 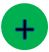 | 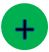 |
| Parisod et al., 2018 | 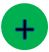 | 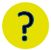 | 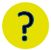 | 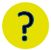 | 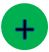 | NA | 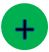 | 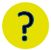 |
| Cremers et al., 2015 | 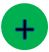 | 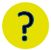 | 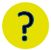 | 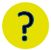 | NA | 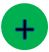 | 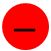 | 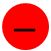 |
| Dietrich et al., 2015 | 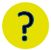 | 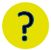 | 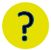 | 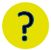 | 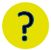 | NA | 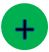 | 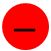 |
| Peskin et al., 2019 | 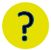 | 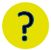 | 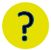 | 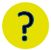 | NA | 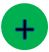 | 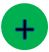 | 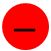 |
| Potter et al., 2016 | 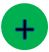 | 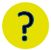 | 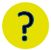 | 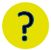 | NA | 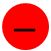 | 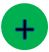 | 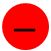 |
| Tortolero et al., 2010 | 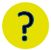 | 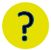 | 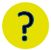 | 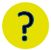 | NA | 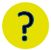 | 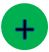 | 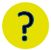 |
| Peskin et al., 2015 | 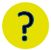 | 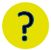 | 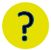 | 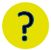 | NA | 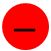 | 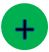 | 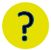 |
| Dcruz, 2014 | 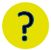 | 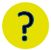 | 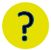 | 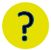 | NA | 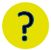 | 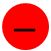 | 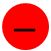 |
| Ismayilova and Terlikbayeva, 2018 |  |  |  |  | NA |  |  |  |
| Sznitman et al., 2011 |  |  |  |  | NA |  |  |  |
| Kaufman et al., 2018^a^ |  |  |  |  |  |  |  |  |
| Lotrean et al., 2010 |  |  |  |  | NA |  |  |  |
| Schwinn et al., 2010 |  |  |  |  |  |  |  |  |
| Cunningham et al., 2009 |  |  |  |  |  |  |  |  |
| Markham et al., 2012 |  |  |  |  | NA |  |  |  |
| Winskell et al., 2018 |  |  |  |  |  | NA |  |  |
| Musiimenta, 2012 |  |  |  |  | NA |  |  |  |

+ = Low risk

- = High risk

? = Unclear risk

NA = not applicable

^a^ Short-term outcomes 0-3 months
