# Supplementary material for: Systematic review of digital interventions to support refusal self-efficacy in child and adolescent health promotion
Source: Health Promot Int. 2022 Sep 27;37(5):daac085. doi: 10.1093/heapro/daac085 (PMC10243864; doi:10.1093/heapro/daac085)
Supplement: daac085_suppl_Supplementary_Table_S2 [file daac085_suppl_supplementary_table_s2.docx]

Table S2. Characteristics of the included studies (n=23) and the effects of the digital health interventions on child and adolescent refusal self-efficacy.

| Authors, publication year, country and intervention name/ short description | Study design and setting | Study sample (I=intervention group, C=control group) | Measured outcome | Instrument | Intervention  (Health topic, theoretical framework, intervention characteristics, duration and intensity) | Control condition | Intervention effects + / 0 | Description | | |  |
| --- | --- | --- | --- | --- | --- | --- | --- | --- | --- | --- | --- |
| Schinke et al.  2009, USA  *A Computer-Mediated, Mother-Daughter Program* | Design not reported (2 groups, random allocation, pretest, posttest & follow-up measures).  Home setting. | n=202  Female 100 %  Mean age: 12.2 years (range 10-13)  Also mothers of the female adolescents. | Alcohol abstinence self-efficacy. | The 20-item Alcohol Abstinence Self-Efficacy Scale. | Alcohol use prevention.  Family Interaction Theory.  Computer program with animated characters, exercises and quizzes, and emails.  14 modules, 4-5 modules each week. | (1) No intervention | + | Compared to the control group (pretest mean 3.59 (SD 0.69), posttest mean 3.52 (SD 0.73), 2 months mean 3.51 (SD 0.69)) the intervention had positive effects on girls’ self-efficacy to avoid alcohol (F = 6.60, p < 0.05, pretest mean 3.63 (SD 0.59), posttest mean 3.72 (SD 0.57), 2 month mean 3.77 (SD 0.47)) at 2-month follow-up and the improvements were greater over time (F = 3.18, p < 0.05). | | |  |
| Fang and Schinke  2014, USA  *A Culturally-Generic, Family-Based Mother-Daughter Intervention* | Randomized control trial.  Home setting. | n=108  Female 100 %  Mean age: 13.10 years (range 10-14)  Also mothers of the female adolescents. | Self-efficacy to avoid substances in tempting situations. | 5 items adapted from the 31-item Self-Efficacy Scale. | Substance use prevention.  Family Interaction Theory.  Computer program with games, simulations and role plays.  9 modules of 30-45 minutes each. | (1) No intervention | + | At 1-year follow-up, intervention-arm girls’ improved family relationships (B = 0.37, p < 0.0001, bias-corrected 95% CI: 0.23, 0.51) were associated with increased self-efficacy (B = 0.38, p < 0.0001). Increased self-efficacy led to decreased alcohol use (B = −0.95, p < 0.01, bias-corrected 95% CI: −1.60, −0.29), marijuana use (B = −0.51, p < 0.05, bias-corrected 95% CI: −0.99, −0.02) and substance use intentions (B = −0.84, p < 0.0001, bias-corrected 95% CI: −1.09, −0.59) at 2-year follow-up. | | |  |
| Fang and Schinke  2013, USA  *A Culturally-Generic, Family-Based Mother-Daughter Intervention* | Randomized control trial.  Home setting. | n=108 Female 100%  Mean age: 13.10 years (range 10-14)  Also mothers of the female adolescents. | Self-efficacy to avoid substances in tempting situations. | 5 items adapted from the 31-item Self-Efficacy Scale. | Substance use prevention.  Family Interaction Theory.  Computer program with activities such as games, simulations and daughter-mother role play.  9 modules of 35-45 minutes each and 1 annual booster session. | (1) No intervention | + | At 2-year follow-up, girls in the intervention group (baseline mean 3.54 (SD 0.66), 1-year mean 3.68 (SD 0.58), 2-year mean 3.62 (SD 0.59)) reported significantly stronger self-efficacy compared to the control group (baseline mean 3.50 (SD 0.80), 1-year mean 3.29 (SD 0.83), 2-year mean 3.52 (SD 0.78)) (F (2, 90) = 4.32, p = 0.016, η^2^ = 0.04). | | |  |
| Fang et al.  2010, USA  *A Culturally-Generic, Family-Based Mother-Daughter Intervention* | Randomized controlled trial.  Home setting. | n=108  Female 100 %  Mean age: 13.25 (I) and 12.99 years (C) (range 11-14)  Also mothers of the female adolescents. | Self-efficacy in the self-change of smoking behavior. | The 31-item Self-Efficacy Scale. | Substance use prevention.  Family Interaction Theory.  Computer program with narration, games, skill demonstration, and daughter-mother exercises.  9 modules of 45 min each. | (1) No intervention | + | | At posttest, girls in the intervention group (baseline mean 3.54 (SE 0.71), posttest mean 3.78 (SE 0.50)) reported greater self-efficacy compared to the control group (baseline mean 3.46 (SE 0.71, posttest mean 3.24 (SE 0.92)) (Wald χ^2^ = 9.73, p = 0.002). |  |  |
| Norris et al.  2013, USA  *DRAMA-RAMA™* | Randomized controlled trial.  1 school (afterschool program). | n=41  Female 100 %  Median age: 12 years (range 11-14) | Peer resistance self-efficacy. | A 6-item scale: two items drawn from the Resistance Self-Efficacy Scale and four items developed for the study. | Peer resistance skills to avoid risky behavior.  Social Cognitive Theory, Communication Competence Model.  Avatar-based Virtual Reality game, class discussions, and group activities.  5 sessions of 45 minutes each (with 15 minutes of gameplay), once a week for 2 weeks. | (1) Comparison game (Wii Dancing with the Stars™) | + / 0 | The intervention had significant positive effects on peer resistance self-efficacy measure at post-test (F = 4.21, p < 0.05, baseline mean 15.8 (SD 0.041), post-test mean (adjusted) 19.8 (SD 0.005)) but not at 2-month follow-up (F = 0.01, p = 0.92, baseline mean 15.8 (SD 0.041), 2 months mean (adjusted) 18.9 (SD 0.010)). | | |  |
| Chang et al.  2018, 2019 Taiwan  *an E-course program* | Repeated-measure design.  10 schools. | n=84  Female 32.6 % (I) and 24.4 % (C)  Mean age: 17.14 ± 0.91 (I) and 17.43 ± 0.14 years (C) | Self-efficacy to resist drug use. | 9 items developed for the study and partly derived from the Drug Use Resistance Self-Efficacy scale (DURSE) on self-efficacy of illegal drug use resistance ). | Substance use prevention.  Theoretical framework not reported.  Computer program with guidance, films, worksheets, homework exercises, discussions, and text messages.  12 sessions of 90 minutes each in 3 months, and after 3 months another 2 sessions of 90 minutes in one month. | (1) Conventional  didactic education | + / 0 | Compared to the comparison group, adolescents in the intervention group made nonsignificant improvements in drug use resistance self-efficacy (β = 0.64, t = 0.45, p = 0.652) at the first and significant improvements at the second posttest (β = 3.47, t = 3.09, p = 0.003). | | |  |
| Parisod et al.  2018, Finland  *Fume* | Three-armed cluster randomized design.  8 schools. | n=151  Female 52.3 %  Median age: 11 years (range 10-13) | Anti-smoking self-efficacy. | The 15-item Anti-smoking Self-efficacy scale (ASSES). | Smoking prevention.  Health literacy.  Mobile game with minigames, a story and information.  20-minute session and 2 weeks of free usage. | (1) Website intervention  (2) No intervention. | 0 | At 2-week post-test, there were no statistically significant changes within the three groups in anti-smoking self-efficacy (p = 0.65, change in groups: intervention group median 0 (95% CI: 0, 1, range: −13, 45), website group median 0 (95% CI: −1, 1, range: −22, 17), control group median 0 (95% CI: 0, 1, range: −18, 7). | | |  |
| Cremers et al.  2015, Netherlands  *Fun without Smokes* | A cluster randomized controlled trial with three study arms.  162 schools. | n=3213  Female 50.59 %  Mean age: 10.36 years (range 10-12) | Self-efficacy to refuse cigarettes. | 10 questions concerning the ability to refuse cigarettes in different situations. | Smoking prevention.  I-Change Model.  Computer program with feedback messages, information, ability to ask questions, videos, games, and prompt messages.  3 feedback messages on 3 days, and 6 prompt messages every year. | (1) Intervention without prompt messages and (2) No intervention | NA | Intervention effects not reported on self-efficacy to refuse cigarettes. | | |  |
| Dietrich et al.  2015, Australia  *Game On: Know Alcohol (GOKA) program* | Cluster randomized controlled design (part of a larger research project).  14 schools. | n=2337  Female 45.8 %  Mean age: 14.5 years (range 14-16) | Drinking refusal self-efficacy. | A 16-item scale drawn from the adolescent versions  of the Drinking Expectancy Questionnaire-Revised and the Drinking Refusal Self-Efficacy Questionnaire-Revised. | Alcohol use prevention.  Theory of Reasoned Action, Experiential Learning Theory.  Online games, practical activities, and a message from research team.  6 modules in one full school-day. | (1) Control condition did not receive the program | 0 | The program had no significant change effects on the self-efficacy measure (F (1, 1012) = 1.819, p = 0.178) at post-test. | | |  |
| Peskin et al.  2019, USA  *It’s Your Game…Keep It Real* | Group randomized controlled trial.  20 schools. | n=2377  Female 54.7 %  Mean age: 12.99 years (SD 0.57) | Self-efficacy to refrain from having sex. | A 6-item scale derived from pilot tested scales to measure self-efficacy to refrain from having sex. | Sexual health.  Social Cognitive Theory, Social Influence Models, Theory of Triadic Influence.  Group-based classroom activities, personalized journaling, individual, tailored, computer-based activities, and parent–child homework activities.  24 lessons of 45 minutes each for 2 school years. | (1) Usual sexual education programs | 0 | At both follow-ups, there were no statistically significant changes in adolescents’ self-efficacy to refrain from having sex between the two groups (8^th^ grade follow-up: effect size = 0.06, unadjusted p = 0.19, adjusted p = 0.58; 9^th^ grade follow-up: effect size = 0.10, unadjusted p = 0.050, adjusted p = 0.15). | | |  |
| Potter et al.  2016, USA  *It’s Your Game…Keep It Real* | Group randomized trial.  24 schools. | n=3143  2487  Female 54 % (I) and 52.5 % (C)  Mean age: 12.7 (I) and 12.8 years (C) | Self-efficacy to refrain from having sex. | A 6-item scale to measure self-efficacy to refrain from having sex. | Sexual health.  Social Cognitive Theory, Social Influence Models, Theory of Triadic Influence.  Computer activities, and group-based classroom activities with journaling, and parent-child activities.  24 lessons of 50 minutes each for 2 years. | (1) Usual sexual education programs | 0 | At both follow-ups, there were no statistically significant differences in self-efficacy to refrain from having sex between the intervention (8^th^ grade follow-up b = 0.01 (95% CI: –0.06, 0.09) estimated effect size = 0.02; 9^th^ grade follow-up b = 0.01 (95% CI: –0.07, 0.08), estimated effect size = 0.01) and control groups. | | |  |
| Tortolero et al.  2010, USA  *It’s Your Game…Keep It Real* | Randomized controlled trial.  10 schools. | n=1307  Female 59.1 %  Mean age: 13.0 years (SD 0.54) | Self-efficacy to refuse sex. | A 7-item scale derived from pilot tested scales to measure self-efficacy regarding refusing sex. | Sexual health.  Social Cognitive Theory, Social Influence Models, Theory of Triadic Influence.  Computer activities, group-based classroom activities, personal journaling, parent-child homework activities.  24 lessons of 45 minutes each for 2 school years | (1) Regular health classes | + / 0 | Compared to the comparison group (8^th^ grade mean 2.97 (SD 0.86), 9^th^ grade mean 3.01 (SD 0.83)), adolescents in the intervention group (8^th^ grade mean 3.07 (SD 0.85); 9^th^ grade mean 3.07 (SD 0.87)) reported greater self-efficacy to refuse sex at 8^th^ grade (difference in adjusted mean = 0.11, p < 0.05), but not at 9^th^ grade follow-up (difference in adjusted mean = 0.08). | | |  |
| Peskin et al.  2015, USA  *It’s Your Game (IYG)-Tech* | Randomized, two-arm nested design.  19 schools. | n=1571  Female 59.0 %  Mean age: 14.3 years (SD 0.59) | Self-efficacy for refusing sex. | A 7-item scale derived from pilot tested scales to measure refusal self-efficacy. | Sexual health.  Social Cognitive Theory, Social cognitive theories.  Computer program with animated narrators as guides, animated scenarios, modeling and skills practice, videos, wall for reflection and personalization, quizzes, information and role play activities.  13 lessons of 35-45 minutes each. | (1) Usual health education | 0 | The intervention had no significant effects on refusal self-efficacy at 9^th^ grade follow-up (beta (difference in  adjusted mean) = .00, SE = .03, 95% CI: –.06, .06). | | |  |
| Dcruz  2014, USA  *It’s Your Game (IYG)-Tech* | Randomized two-arm nested design.  19 schools. | n=984  Female 57.6 %  Mean age: 15.76 years (range 14-18) | Refusal self-efficacy related to sexual behaviors. | 12 items that represent refusal self-efficacy related to sexual behaviors. | Sexual health.  Social Cognitive Theory.  Computer program with activities.  13 lessons of 35 minutes each. | (1) Usual care | NA | Intervention effects not reported on refusal self-efficacy. | | |  |
| Ismayilova and Terlikbayeva  2018, Kazakhstan  *Kazakhstani Family Together (KFT)* | Mixed methods approach with a randomized control design with three waves of data.  Home / field office setting. | n=181  Female 38.7 %  Mean age: 15.27 years  (range 14-17)  Also caregivers of the adolescents | Substance use resistance and peer and partner refusal skills. | 8 items adapted from the Self-Efficacy for Limiting Substance Use Scale. | Substance use and HIV prevention.  Integrated Behavioral Model, Family Interaction Theory.  Computer platform with communication with an avatar, skill demonstration, exercises and behavioral rehearsal.  3 sessions, of 25-30 minutes each, delivered weekly. | (1) Usual care | 0 | There were no statistically significant differences in resistance self-efficacy between treatment group (baseline mean 3.54 (SD 0.66), 3 months mean 3.72 (SD 0.60), 6 months mean 3.57 (SD 0.78)) and control group (baseline mean 3.48 (SD 0.75), 3 months mean 3.55 (SD 0.75), 6 months mean 3.68 (SD 0.61)) at both post-tests (3 months: Cohen’s d = 0.15, p = 0.411; 6 months: Cohen’s d = –0.25, p = 0.162). | | |  |
| Sznitman et al.  2011, USA  *A mass media intervention* | Randomized trial.  4 cities. | n=1710  1346  Female 56.7 %  Mean age: 15 years (range 14-17) | Sex-refusal self-efficacy. | 6 items drawn from the Sex Refusal Self-Efficacy Scale and the perceived difficulties of performing AIDS preventive behavior scale. | Sexual health.  Social Cognitive Theory.  Television and radio ads.  30-second television and 60-second radio ads running at a constant rate throughout the 16-month recruitment and 18-month follow-up periods. | (1) No intervention | + / 0 | Based on a statistical model, the intervention improved adolescents’ sex-refusal self-efficacy among sexually experienced adolescents (B = −0.15, p = 0.047). | | |  |
| Kaufman et al.  2018, USA  *Multimedia Circle of Life (mCOL)* | Two-armed cluster randomized controlled trial.  6 Native Boys and Girls Clubs. | n=167  Female 52.7 %  Mean age: 11.2 years (range 10-12) | Self-efficacy to resist peer pressure and to avoid sexual risk. | A 3-item scale adapted from the Self-Efficacy  Instrument for Protective Sexual Behaviors to measure self-efficacy to resist peer pressure and a 3-item scale to measure self-efficacy to avoid sexual risk. | Sexual health.  Social Cognitive Theory, Theory of Reasoned Action.  Computer program with stories, games and videos, and group classes with discussions, instructions, demonstrations, games and craft activities.  7 chapters including 20-25-minute online sessions and 1-hour group classes per each chapter. | (1) After-School Science Plus program | + / 0 | Compared to the control group, adolescents in the intervention group reported significantly higher self-efficacy to resist peer pressure (b = −0.41 (SE = 0.17), p = 0.027, (95% CI: −0.77, −0.05)) and avoid sex (b = −1.02 (SE = 0.17), p < 0.001, (95% CI: − 1.39, −0.64)) at posttest, but not at 9-month follow-up (resist peer pressure b = −014 (SE = 0.18), p = 0.449, (95% CI: −0.53, −0.25) and avoiding sex b = 0.17 (SE = 0.26), p = 0.540, (95% CI: −0.40, 0.73)). | | |  |
| Lotrean et al.  2010, Romania  *A peer-led smoking prevention programme* | Study design not reported (2 groups, random allocation, baseline & follow-up measures).  20 schools (55 classes from the 7th grade). | n=1196  Female 50.9 % (I) and 51.5 % (C)  Mean age: 13.7 years (range 13-14) | Self-efficacy in  refraining from smoking. | 12 questions concerning the ability to refrain from cigarettes in social situations, when under emotional strains, and in different routine situations. | Smoking prevention.  Social Cognitive Theory, Social cognitive theories, Integrated Model of Change, Principles of the social influence approach.  Videos including feedback, small group activities, and home activities.  5 weekly sessions of 45 minutes each. | (1) Control condition not described | + / 0 | Compared to the control group (mean −0.07), students in the experimental group (mean 0.07) reported significantly greater social self-efficacy at post-test (p < 0.05, effect size = 0.07), but not emotional nor situational self-efficacy. | | |  |
| Schwinn et al.  2010, USA / Canada  *RealTeen* | Design not reported (2 groups, random allocation, baseline, posttest & follow-up measures).  Home setting. | n=236  Female 100 %  Mean age:  14 years (range 13-14) | Self-efficacy to resist pressure to use substances. | A 4-item scale created for the study to measure girls’ ability to resist pressure from a boyfriend or a boy one likes to use substances. | Substance use prevention.  Social Learning Theory.  Website with personalization options, pen pals, animated characters as guides, lessons, exercises, news, horoscopes, beauty tips, blogs, chat forums and diaries.  12 sessions of 25 minutes each, 2 sessions per week. | (1) No intervention | + / 0 | At 6-month follow-up but not at 6-week posttest, girls in the intervention group reported greater self-efficacy compared to the control group (6-month follow-up: F (1,193) = 4.19, p < 0.05). | | |  |
| Cunningham et al.  2009, USA  *SafERteens* | Randomized controlled trial.  1 medical center. | n=533  Female 58.2 %  Mean age: 16.7 years (range 14-18) | Self-efficacy related to not drinking alcohol and nonviolence. | 5 items to measure self-efficacy to say no to drinking alcohol drawn from the Specific Event Drug and  Alcohol Refusal Efficacy measure, and the 5-item Self-Efficacy—Teen Conflict Survey to measure self-efficacy for nonviolence. | Alcohol use and violence prevention.  Principles of motivational interviewing.  Computer program with narration, virtual buddies guiding and giving feedback, interaction with peers and behavioral exercises.  35-minute brief intervention. | (1) Brief intervention delivered by a therapist  (2) Control (received an information brochure) | + / 0 | Compared to the control group (baseline mean 2.32 (SD 1.21), 3 months mean 2.38 (SD 1.34)), adolescents’ in the computer intervention group reported increased self-efficacy related to not drinking alcohol at posttest (baseline mean 2.25 (SD 1.22), posttest mean 2.47 (SD 1.07), p ≤ 0.05) but not at the 3-month follow-up (mean 2.49 (SD 1.35), p = 0.083)). Results for the therapist intervention were similar but the therapist intervention group reported increased non-alcohol self-efficacy also at the follow-up (baseline mean 2.14 (SD 1.18), posttest mean 2.46 (SD 1.16), p ≤ 0.01; 3 months mean 2.51 (SD 1.32), p = 0.050). Compared to the control group (baseline mean 2.44 (SD 0.85), 3 months mean 2.53 (SD 0.84)), adolescents in the computer intervention group reported increased self-efficacy related to nonviolence a posttest (baseline mean 2.41 (SD 0.84), posttest mean 2.70 (SD 0.92), p ≤ 0.01) and at the 3-month follow-up (mean 2.73 (SD 0.83), p = 0.002). Results for the therapist intervention were similar (baseline mean 2.24 (SD 0.79), posttest mean 2.65 (SD 0.81), p < 0.001; 3 months mean 2.51 (SD 0.87), p = 0.041) | | |  |
| Markham et al.  2012, USA  *Sexual Risk Avoidance (RA) and Sexual Risk Reduction (RR) Interventions* | Three-armed, randomized controlled trial.  15 schools. | n=1258  Female 59.8 %  Mean age: 12.6 years (SD 0.76) | Self-efficacy to refuse sex. | A 7-item scale to measure self-efficacy regarding refusing sex. | Sexual health.  Social Cognitive Theory, Theory of Planned Behavior.  Computer activities (e.g. skills training, videos, teen serials, feedback), group-based classroom activities, journaling, parent-child homework activities.  24 lessons of 50 minutes each for 2 school years. | (1) Risk avoidance program (similar activities but targeting abstinence education)  (2) regular health classes | + / 0 | Relative to the control group, adolescents in the risk reduction program but not in the risk avoidance program reported significantly greater refusal self-efficacy at 8^th^ grade (Risk reduction program: beta coefficient = 0.11, p < 0.01; Risk avoidance program: beta coefficient = 0.07), but not at 9^th^ grade follow-up (Risk reduction program: beta coefficient = 0.02; Risk avoidance program: beta coefficient = 0.01). | | |  |
| Winskell et al.  2018, Kenya  *Tumaini* | Randomized controlled trial.  Home setting. | n=60  Female 50 %  Mean age: 12.7 years (range 11-14) | Self-efficacy for sexual risk avoidance. | A 9-item scale drawn from the instruments used in the Families Matter! Program (FMP), Save the Children’s questionnaire, Johnson-Mallard’s assessment of women’s self-efficacy, Fisher et al’s IMB subscales | Sexual health.  Social Cognitive Theory, Theory of Possible Selves.  Smartphone game including a role-playing narrative, mini-games, and a goal-setting and reflection exercise.  1-hour gameplay per day for 16 days. | (1) No intervention (standard of care) | + | The intervention arm adolescents (immediately post-intervention mean 1.95 (SD 1.57), 6 weeks mean 2.03 (SD 1.83)) reported significantly higher self-efficacy compared to the control arm (immediately post-intervention mean 0.47 (SD 1.07), 6 weeks mean 0.63 (SD 1.20)) at immediately (p < 0.001) and 6 weeks post-intervention (p < .001, t_58_ = −3.50). | | |  |
| Musiimenta  2012, Uganda  *World Starts With Me (WSWM)* | Controlled pre-post intervention evaluation.  2 schools. | n=300  Female 58 % (I) and 68 % (C)  Age range: 11-16 years (73.3 % of the participants 14-16 years) | Condom assertiveness self-efficacy. | 5 statements from the Sexual Assertiveness Scale (SAS) to assess girls’ perceived condom assertiveness self-efficacy | Sexual health.  Theoretical framework not reported.  Computer program and website with online counselling, support centre, virtual peer educators, quizzes, story boards and role plays.  14 lessons. | (1) No intervention | + | At post-test, girls in the intervention group reported significantly improved ability to refuse having unprotected sex (pre-test mean 2.16, post-test mean 1.55, SD 0.96, p = 0.03) compared to the comparison group (pre-test mean 2.18, post-test mean 2.20, SD 0.15, p = 0.16) (between-group pre-test p = 0.32, post-test p = 0.00). | | |  |
| + = statistically significant favorable results  0 = statistically nonsignificant results  + / 0 = both statistically significant favorable results and statistically nonsignificant results on refusal self-efficacy at different time points, with different subgroups, or domains of refusal self-efficacy  NA = not available, CI = Confidence interval, SD = standard deviation, SE = standard error | | | | | | | | | | | |
