# Supplementary material for: Systematic review of digital interventions to support refusal self-efficacy in child and adolescent health promotion
Source: Health Promot Int. 2022 Sep 27;37(5):daac085. doi: 10.1093/heapro/daac085 (PMC10243864; doi:10.1093/heapro/daac085)
Supplement: daac085_suppl_Supplementary_Table_S3 [file daac085_suppl_supplementary_table_s3.docx]

Table S3. Study-specific findings: digital interventions to support child and adolescent refusal self-efficacy.

| Authors | Measured refusal self-efficacy outcomes | Questionnaires | Digital intervention group | Control group (no intervention) | Control group (another intervention) | Between group results | P-value |  |
| --- | --- | --- | --- | --- | --- | --- | --- | --- |
| Schinke et al., 2009 | Substance use refusal self-efficacy | 20 self-report items on a 5-point scale (not at all confident – extremely confident) | T0 mean: 3.63  (SD 0.59)  T1 mean: 3.74  (SD 0.57)  T2 mean: 3.77  (SD 0.47) | T0 mean: 3.59  (SD 0.69)  T1 mean: 3.52  (SD 0.73)  T2 mean: 3.51  (SD 0.69) |  | Time x intervention interaction:  F = 3.18  η^2^ = 0.02 | **< 0.05*** |  |
| Fang and Schinke, 2013, 2014 | Substance use refusal self-efficacy | 5 self-report items on a 5-point scale (not at all sure – extremely sure) | T0 mean: 3.54  (SD 0.66)  T1 mean: 3.68  (SD 0.58)  T2 mean: 3.62  (SD 0.59) | T0 mean: 3.50  (SD 0.80)  T1 mean: 3.29  (SD 0.83)  T2 mean: 3.52  (SD 0.78) |  | Time x intervention interaction:  F = 4.32  η^2^ = 0.04 | **0.016*** |  |
| Fang et al., 2010 | Substance use refusal self-efficacy | 31 self-report items on a 5-point scale (higher scores are better) | T0 mean: 3.54  (SD 0.69)  T1 mean: 3.78  (SD 0.50) | T0 mean: 3.46  (SD 0.71)  T1 mean: 3.24  (SD 0.92) |  | Time x intervention interaction:  Wald χ2 = 9.73 | **0.002*** |  |
| Chang et al., 2018, 2019 | Substance use refusal self-efficacy | 9 self-report items on a 4-point scale (not sure at all – definitely sure) | T0 score: 31.55  T1 score: 32.65  T2 score: 35.87 | T0 score: 34.14  T1 score: 34.59  T2 score: 34.99 |  | T1:  β = 0.64 (SE 1.42)  t = 0.45  T2:  β = 3.47 (SE 1.12)  t = 3.09 | T1: 0.652  T2: **0.003*** |  |
| Parisod et al., 2018 | Substance use refusal self-efficacy | 15 self-report items on a 4-point scale (can’t do at all – certainly can do) | T0 median: 55  (95% CI: 53, 56)  T1 median: 57  (95% CI: 53, 58) | T0 median: 57  (95% CI: 54, 58)  T1 median: 56  (95% CI: 55, 58) | T0 median: 56  (95% CI: 54, 57)  T1 median: 56  (95% CI: 53, 58) |  | 0.46 |  |
| Dietrich et al., 2015 | Substance use refusal self-efficacy | 16 self-report items on a 6-point scale (very sure I could not – very sure I could) | T0 score (abstainers):  84.5 (SD 16.9)  T1 score (abstainers):  81.8 (SD 22.2)  T0 score (bingers):  63.2 (SD 22.1)  T1 score (bingers):  68.4 (SD 20.5)  T0 score (moderate drinkers): 75.8  (SD 18.0)  T1 score (moderate drinkers):  74.1 (SD 23.2) | T0 score (abstainers):  83.0 (SD 19.2)  T1 score (abstainers):  79.3 (SD 24.4)  T0 score (bingers):  64.7 (SD 19.5)  T1 score (bingers):  64.3 (SD 21.7)  T0 score (moderate drinkers): 77.3  (SD 17.1)  T1 score (moderate drinkers):  75.0 (SD 21.7) |  | Time x intervention interaction:  F(1, 1012) = 1.819 | 0.178 |  |
| Ismayilova and Terlikbayeva, 2018 | Substance use refusal self-efficacy | 8 self-report audio computer-assisted self-interview items on a 4-point scale (very easy – very hard) | T0 mean: 3.54  (SD 0.66)  T1 mean: 3.72  (SD 0.60)  T2 mean: 3.57  (SD 0.78) | T0 mean: 3.48  (SD 0.75)  T1 mean: 3.55  (SD 0.75)  T2 mean: 3.68  (SD 0.61) |  | Effect size:  T1: Cohen’s d = 0.15  T2: Cohen’s d = -0.25 | T1: 0.411  T2: 0.162 |  |
| Lotrean et al., 2010 | Substance use refusal self-efficacy | 12 self-report items on a 7-point scale (sure I will smoke [-3] – sure I won’t smoke [+3]) | Adjusted mean at T1 (corrected for T0 scores):  Social self-efficacy: 0.07  Emotional self-efficacy: 0.06  Situational self-efficacy: 0.05 | Adjusted mean at T1 (corrected for T0 scores):  Social self-efficacy: -0.07  Emotional self-efficacy: -0.05  Situational self-efficacy: -0.05 |  | Effect size:  Social self-efficacy: 0.07  Emotional self-efficacy: 0.05  Situational self-efficacy: 0.05 | Social self-efficacy:  **< 0.05***  Emotional self-efficacy: non-significant  Situational self-efficacy: non-significant |  |
| Schwinn et al., 2010 | Substance use refusal self-efficacy | 4 self-report items on a 4-point Likert scale | Not reported | Not reported |  | Time x intervention interaction:  T2: F(1, 193) = 4.19 | T1: non-significant  T2: **< 0.05*** |  |
| Cunningham et al., 2009 | Substance use refusal self-efficacy | 5 self-reported audio computer-assisted self-interview items on a 5-point scale (not at all sure – extremely sure) | T0 mean: 2.25  (SD 1.22)  T1 mean: 2.47  (SD 1.07)  Difference in mean: 0.18 (SD 1.11)  % Change in mean: 8.00 (p ≤ 0.05)  T2 mean: 2.49  (SD 1.35) | T0 mean: 2.32  (SD 1.21)  T2 mean: 2.38  (SD 1.34) | T0 mean: 2.14  (SD 1.18)  T1 mean: 2.46  (SD 1.16)  Difference in mean: 0.28 (SD 0.99)  % Change in mean: 13.08 (p ≤ 0.01)  T2 mean: 2.51  (SD 1.32)  Effect size: 0.20  (p = 0.05) |  | Digital intervention group: 0.083 |  |
|  | Violence refusal self-efficacy | 5 self-reported audio computer-assisted self-interview items on a 5-point scale (not at all sure – extremely sure) | T0 mean: 2.41  (SD 0.84)  T1 mean: 2.70  (SD 0.92)  Difference in mean: 0.29 (SD 0.72)  % Change in mean: 12.03 (p ≤ 0.01)  T2 mean: 2.73  (SD 0.83)  Effect size: 0.31  (p = 0.002) | T0 mean: 2.44  (SD 0.85)  T2 mean: 2.53  (SD 0.84) | T0 mean: 2.24  (SD 0.79)  T1 mean: 2.65  (SD 0.81)  Difference in mean: 0.41 (SD 0.70)  % Change in mean: 18.30 (p ≤ 0.001)  T2 mean: 2.51  (SD 0.87)  Effect size: 0.22  (p = 0.041) |  | Digital intervention group: **0.002*** |  |
| Peskin et al., 2019 | Sex refusal self-efficacy | 6 self-report (audio-enhanced,  computer-assisted) items on a 4-point scale (definitely could not – definitely could) | Not reported | Not reported |  | Effect size:  T1: 0.06  T2: 0.10 | T1: 0.58  T2: 0.15 |  |
| Potter et al., 2016 | Sex refusal self-efficacy | 6 self-report audio-enhanced,  computer-assisted items on a 4-point scale (no, I definitely could not – yes, I definitely could) | Not reported | Not reported |  | T1: β = 0.01,  95% CI: -0.06, 0.09  Effect size: 0.02  T2: β = 0.01,  95% CI: -0.07, 0.08  Effect size: 0.01 | T1: non-significant  T2: non-significant |  |
| Tortolero et al., 2010 | Sex refusal self-efficacy | 7 self-report audio computer-assisted self-interview items on a 4-point scale (definitely could not – definitely could) | T0 mean: 3.09  (SD 0.81)  T1 mean: 3.07  (SD 0.85)  T2 mean: 3.07  (SD 0.87) | T0 mean: 3.14  (SD 0.77)  T1 mean: 2.97  (SD 0.86)  T2 mean: 3.01  (SD 0.83) |  | Difference in adjusted means:  T1: 0.11  T2: 0.08 | T1: **< 0.05***  T2: non-significant |  |
| Peskin et al., 2015 | Sex refusal self-efficacy | 7 self-report audio computer-assisted self-interview items on a 4-point scale (range of scores 1 – 4) | Not reported | Not reported |  | β = 0.00 (SE 0.03),  95% CI: -0.06, 0.06 | non-significant |  |
| Sznitman et al., 2011 | Sex refusal self-efficacy | 6 self-report audio computer-assisted self-interview items: 4 items on a 4-point scale (definitely could – definitely could not), and 2 items on a 6-point scale (very easy – very hard) | Not reported | Not reported |  | β = 0.04  Sexually experienced:  β = -0.15 | 0.551  Sexually experienced:  **0.047*** |  |
| Markham et al., 2012 | Sex refusal self-efficacy | 7 self-report audio computer-assisted self-interview items on a 4-point scale (definitely could not – definitely could) | Not reported | Not reported | Not reported | Risk reduction intervention:  T1: β = 0.11  T2: β = 0.02  Risk avoidance intervention:  T1: β = 0.07  T2: β = 0.01 | Risk reduction intervention:  **T1: < 0.01***  T2: non-significant  Risk avoidance intervention:  T1: non-significant  T2: non-significant |  |
| Winskell et al., 2018 | Sex refusal self-efficacy | 9 self-report audio computer-assisted  self-interview items on a 3-point scale (very sure – not sure) | T0 score: 5.87  (SD 2.03)  T0 – T1 change in score: 1.95 (SD 1.57)  T0 – T2 change in score: 2.03 (SD 1.83) | T0 score: 6.22  (SD 2.41)  T0 – T1 change in score: 0.47 (SD 1.07)  T0 – T2 change in score: 0.63 (SD 1.20) |  |  | T1: **< 0.001***  T2: **< 0.001*** |  |
| Musiimenta, 2012 | Sex refusal self-efficacy | 5 self-report items on a 4-point scale (1 item on sex refusal self-efficacy) (strongly agree – strongly disagree) | T0 mean: 2.16  T1 mean: 1.55  SD = 0.96  (p = 0.03) | T0 mean: 2.18  T1 mean: 2.20  SD = 0.15  (p = 0.16) |  |  | **0.00*** |  |
| Kaufman et al., 2018 | Sex refusal self-efficacy | 3 self-report items on a 4-point scale (definitely would not do – definitely would do) | T1 mean: 2.19  (SD 1.24)  T2 mean: 2.04  (SD 1.25) |  | T1 mean: 2.60  (SD 1.29)  T2 mean: 2.67  (SD 1.27) | T1: β = -1.02 (SE 0.17),  95% CI: -1.39, -0.64  T2: β = 0.17 (SE 0.26),  95% CI: -0.40, 0.73 | T1: **0.000***  T2: 0.540 |  |
|  | Peer resistance self-efficacy | 3 self-report items on a 4-point scale (definitely would not – definitely would) | T1 mean: 3.78  (SD 0.56)  T2 mean: 3.60  (SD 0.71) |  | T1 mean: 3.60  (SD 0.77)  T2 mean: 3.65  (SD 1.27) | T1: β = -0.41 (SE 0.17),  95% CI: -0.77, -0.05  T2: β = -0.14 (SE 0.18),  95% CI: -0.53, -0.25 | T1: **0.027***  T2: 0.449 |  |
| Norris et al., 2013 | Peer resistance self-efficacy | 6 self-report items on a 5-point scale (not at all sure – completely sure) | T0 score: 15.8  (SD 0.041)  T1 score: 19.8  (SD 0.005)  T2 score: 18.9  (SD 0.010) |  | T0 score: 16.8  (SD 0.034)  T1 score: 17.9  (SD 0.006)  T2 score: 19.0  (SD 0.012) | Time x intervention interaction:  T1: F = 4.21  T2: F = 0.01 | T1: **< 0.05***  T2: 0.92 |  |
| * = Statistically significant at 0.05 significance level  SD = standard deviation, SE = standard error, CI = confidence interval | | | | | | | | |
